# Gender Differences in the Impact of Abuse and Neglect Victimization on Adolescent Offending Behavior

**DOI:** 10.1007/s10896-014-9668-4

**Published:** 2015-01-25

**Authors:** Jessica J. Asscher, Claudia E. Van der Put, Geert Jan J. M. Stams

**Affiliations:** Forensic Child and Youth Care Sciences, University of Amsterdam, Nieuwe Achtergracht 127, POBox 15776, 1001 NG Amsterdam, The Netherlands

**Keywords:** Abuse and neglect victimization, Adolescent offending, Transmission of antisocial behavior, Gender differences

## Abstract

The present study examines gender differences in the association between abuse and neglect during childhood, and sexual and violent offending in juvenile delinquents. Female juvenile delinquents were more frequently victim of sexual and physical abuse and had a history of neglect and maltreatment than male juvenile offenders. Male juvenile offenders committed more sexual offenses and felony offenses against persons. Female juvenile offenders reported higher levels of having committed misdemeanor offenses against persons and violence that were not included in criminal history. A history of sexual abuse was related to sexual offending, while a history of physical abuse was related to violent offending. The relationships between victimization and offending were stronger in male juvenile offenders than in female juvenile offenders.

There is an ample body of research demonstrating the negative consequences of maltreatment on child development (Thornberry et al. 2012). Child maltreatment can include emotional abuse, neglect, sexual abuse, physical abuse, or any combination thereof (Thornberry et al. 2012). It is often believed that being a victim of child maltreatment is a risk factor for being a perpetrator of maltreatment later in life, which is referred to as the ‘cycle of violence’ (Widom 1989a). Specifically, there is empirical evidence showing that child maltreatment is associated with an increased likelihood of becoming a juvenile delinquent (Widom and Maxfield 2001). It, therefore, seems important to study (intergenerational) transmission of antisocial behavior.

In particular, abuse experiences early in life are likely to have an impact during the rest of the lives of the victims, because, for instance, victims may experience post-traumatic stress symptoms (Kearney et al. 2010). Behaviorist theories suggest that by experiencing maltreatment, children learn that hurting and harming others is ‘normal’ (Bandura 1973; 1977). The maltreating behavior is modeled and internalized. Consequently, children are likely to repeat such behaviors (Bandura and Ribes-Inesta 1976). Alternatively, theories of lifestyle and routine activity assume that becoming a perpetrator after having been victimized results from lack of parental supervision, with more exposure to offender populations as a consequence (Cohen et al. 1981).

Kerig and Becker 2010 describe various theoretical models explaining the association between traumatic childhood experiences and delinquency. The development of delinquent behavior can be explained from an emotion regulation perspective (i.e., affect dysregulation, emotional numbing, emotion recognition). Emotion regulation capacities may be impaired by chronic and pervasive maltreatment. In this case, the development of delinquent behavior can be explained by increased irritability and impulsivity, emotions are processed differently by abused children than by non-maltreated children, and emotions are suppressed. An alternative explanation that Kerig and Becker provide lies in cognitive processes, such as hostile attribution, stigmatization, and alienation, which is a process of moral disengagement. Abused children tend to interpret environmental signals negatively, and are relatively sensitive to rejection, which expresses itself in a more intense reaction to and anticipation of rejection.

Another cognitive problem abused children experience is the inability to recognize and respond to risks, which may precede aggression or violence. From this perspective delinquency may be seen as a coping strategy in response to a violent environment.

Interpersonal processes may also explain the association between maltreatment and delinquency. For example, abuse victimization may result in affiliation with deviant peers (Bolger et al. 1998), which has been shown to be related to delinquent behavior (Barnow et al. 2005; Gifford-Smith et al. 2005). Negative parent-child relationships, in terms of maltreatment and harsh parenting, have been linked with development of delinquent behavior (Asscher et al. 2013; Hoeve et al. 2009). Finally, attachment problems, as a consequence of abuse (Crittenden and Ainsworth 1989), may be predictive of delinquent behavior (Hoeve et al. 2012).

Although neglect is less studied than abuse, results of the limited research available suggest, in line with the findings for abuse, that neglect is associated with the development of severe behavioral problems. For example, boys who were victim of childhood neglect were convicted for offending four times more than juveniles who were not exposed to childhood neglect (Kazemian et al. 2011). There are indications that specific victimization types predict the same kind of offending behavior (e.g., Ford and Linney 1995). However, not all studies reported these relations (e.g., Epps et al. 1996).

Despite the long tradition of research into the associations between maltreatment and the development of delinquency, many topics remain understudied (George 2012). For example, the impact of maltreatment in specific subgroups has not been well-studied (Mersky and Reynolds 2007). For instance, the differential effect of maltreatment on the development of psychopathology for boys and girls has been a neglected topic in the literature.

Studies that provide information on potential gender differences in the effects of maltreatment are inconsistent (George 2012). (Topitzes et al. 2011) found evidence for gender specific relations between child maltreatment and delinquency. Child maltreatment predicted juvenile delinquency in males, but not in females. However, child maltreatment did predict adult crime for both genders, which suggests that the effects on delinquent behavior of child maltreatment may be a bit delayed in girls (Topitzes et al. 2011). Moreover, different additional childhood risk factors for delinquency were visible. Different risk factors mediated the maltreatment-crime relation in females and males. For males, childhood externalizing behaviors, school commitment, social-emotional skills, and educational attainment during adolescence affected the relation between maltreatment and crime, whereas parental factors in childhood and externalizing problems, cognitive performance, mobility, and educational attainment in adolescence affected this relation in females (Topitzes et al. 2011). Other researchers have reported that, the consequences of maltreatment, such as trauma, play a greater role in the development of delinquent behavior in females than in males (Foy et al. 2012; Hipwell and Loeber 2006; McCabe et al. 2002). However, they did not examine victims of sexual abuse.

The fact that there are many different forms of maltreatment may explain the mixed results on gender differences in the association between child maltreatment and delinquency. Due to sample size problems, the many forms of maltreatment are often combined in analyses, which may result in different results than when these forms of maltreatment are analyzed separately. There are indications that different types of maltreatment are more prevalent in boys and girls. For example, girls are more likely to be sexually abused by family members than boys (Finkelhor et al. 1990), Girls are also more likely to have experienced penetrative abuse (Kohn Maikovich-Fong and Jaffee 2010). Boys are believed to be physical abuse victims more often than girls (Titus et al. 2003). Consequently the association between child maltreatment and delinquency may be different in boys and girls.

In order to be able to provide adequate treatment for juvenile delinquency, it is thus important to distinguish different types of maltreatment, as types of victimization may be gender specific, causing gender differences in offending . The present study aims to add to the literature by examining 1) whether there are gender differences in type of abuse experienced; 2) whether there are gender differences in type of offenses committed; and 3) whether the association between maltreatment and sexual and violent offending is different in male and female juvenile offenders. This knowledge can be used to improve treatment of abused juvenile offenders by addressing their criminogenic needs more specifically.

## Method

### Sample

For this study, data from the Washington State Juvenile Court Assessment (WSJCA) validation study were used (Barnoski 2004), consisting of American juveniles, aged 12 to 18, who were found guilty of a criminal act by a juvenile court and for whom the WSJCA was completed. Data were available for female (*n* = 3,502) and male (*n* = 10,111) juveniles. Sixty-one percent of the females (*n* = 2,136) and 37 % of the males (*n* = 3,741) had experienced some form of maltreatment.

### Instruments and Procedure

#### Washington State Juvenile Court Assessment (WSJCA)

The WSJCA is a screening and risk assessment instrument, which was developed in Washington State (Barnoski 2004). The WSJCA maps out the most important risk and protective factors in a large number of domains. The selection of domains and items took place on the basis of a review of the juvenile delinquency research literature and then was modified, based on feedback from an international team of experts. The assessment was revised again following reviews by Washington State juvenile court professionals.

The WSJCA comprises two parts: a pre-screen and full assessment. The pre-screen is a shortened version of the full assessment that quickly indicates whether a youth is at low, moderate or high risk for reoffending. It comprises the most important predictors of recidivism from two domains: the criminal history domain and the social history domain (Barnoski 2004). Additionally, the pre-screen is administered to all youth on probation and the full assessment is required only for youth who are assessed as having moderate or high risk on the pre-screen (71 % of the juvenile offenders). The full assessment identifies a youth’s risk and protective factor profile to guide rehabilitative efforts. The courts have refocused their resources on moderate and high risk youth by assigning low risk youth to minimum supervision caseloads.

Trained probation officers perform the full assessments on the basis of information from a structured motivational interview with the youth and youth’s family. This training includes reviewing video-taped interviews and the resulting assessment to ensure that the probation officer has mastered the assessment skills. There is a manual available for the full assessment, and quality assurance is an important part of the assessment structure and organization in Washington State (Barnoski 2004). The quality assurance consists of a training manual and curriculum, which ensures that the staff completing the assessments understand the concepts intended to be assessed. Assessment staff members are trained and have received feedback to become certified trainers. Additionally, the items concerning schools (e.g., grades) were checked with the schools which the juveniles were attending.

The predictive validity of the WSJCA has been tested in three studies (Barnoski 2004; Orbis Partners Inc 2007; Van der Put et al. 2012). In the first study conducted by Barnoski, the Area Under the receiver-operating-characteristics Curve (AUC) of the WSJCPA was .64 and in the last two studies the AUC was .63. In a meta-analysis of the predictive validity of risk-assessment instruments for juveniles, it was shown that the AUC varied from 0.53 to 0.78, with an average AUC of 0.64 (Schwalbe, 2007). The AUC of the WSJCPA is, therefore, comparable to the average AUC of juvenile justice risk assessment instruments.

In the present study, we analysed the types of offense committed by males and females. Additionally, we compared the abuse history of males and females. Finally, we examined the relation between abuse history and recidivism.

### History of Abuse/Neglect

Juveniles were asked to report on the histories of physical abuse, sexual abuse and neglect. The self-reported information was checked with child protective services, community mental health, and other sources of information. Any history of being a victim of physical or sexual abuse or neglect that was suspected, whether or not substantiated, was included. This risk factor indicates suspected abuse that may or may not be confirmed. Reports of abuse or neglect that proved to be false were excluded (Washington State Institute for Public Policy 2004).

*Physical abuse* was assessed by asking the juveniles to indicate whether they were a victim of physical abuse, and if they indicated to be so, they were asked to indicate if they were physically abused by a family member or physically abused outside the family. Child Protective Services define physical abuse as any non-accidental physical injury, such as bruises, burns, fractures, bites, or internal injuries (Washington State Institute for Public Policy 2004).

*Sexual abuse* includes acts such as indecent liberties, communication with a minor for immoral purposes, sexual exploitation of a child, child molestation, sexual misconduct with a minor, rape of a child, and rape (Washington State Institute for Public Policy 2004). In the present study, like physical abuse, juveniles were asked to report on their sexual abuse history by indicating whether they were a victim of sexual abuse, and if so, whether they were sexually abused by a family member or if they were sexually abused outside the family.

*Neglect* was assessed by using the Child Protective Services’ (CPS) definition of neglect, which includes negligence, maltreatment (dangerous act), or omission that constitutes a clear and present danger to the child’s health, welfare, and safety. This includes: failure to provide adequate food, clothing, shelter, emotional nurturing, or health care; failure to provide adequate supervision in relation to the child’s level of development; an act of abandonment with the intent to forego parental responsibilities despite an ability to do so; an act of exploitation, such as requiring the child to be involved in criminal activity, imposing unreasonable work standards, etc.; an act of reckless endangerment, such as a parent driving under the influence of alcohol or drugs with children present; and other dangerous acts, such as hitting, kicking, throwing, choking a child, or shaking an infant (Washington State Institute for Public Policy 2004). Like abuse, juveniles were asked to report on their neglect history by indicating on a dichotomous scale if they were victim of neglect.

For a more elaborate description of how juveniles were asked to report on their abuse histories, please find the section concerning abuse history from the research protocol in Apendix 1.

### Criminal History, Reports of Violence not included in Criminal History

In order to assess criminal history, age of first offense, sexual misconduct misdemeanour referrals and felony sex offense referrals were coded. The criminal history variable is based on official records of delinquent behaviour. All felony and misdemeanour referrals that resulted in a conviction, deferred adjudication, or a deferred disposition were summed, separately coded for weapon referrals, against person misdemeanour referrals, and against person felony referrals. Finally, disposition orders where youth served at least one day confined in detention, disposition orders where youth served at least one day in confined under Juvenile Rehabilitation Authority (JRA), escapes and failure to appear in court warrants were assessed.

In order to assess evidence of violence that is not included in criminal history, information that is provided by the school, family, juvenile him/herself and other professionals involved was examined. The following items were assessed: violent outbursts, displays of temper, uncontrolled anger indicating potential for harm, deliberately inflicting physical pain, using/threatening with a weapon, fire starting, violent destructions of property, and animal cruelty.

### Analyses

To answer the first research question, whether there are gender differences in type of abuse experienced, percentages of type of offense, and percentages of different types of experienced maltreatment were compared for males and females by means of a series of chi-square tests. T-tests were used to determine whether there were mean differences between males and females in total maltreatment. Percentages of type of offense and type of maltreatment experienced were compared with chi square tests.

The association between the different types of maltreatment and recidivism were calculated separately for males and females (research question 3). Fischer Z tests were conducted to detect significant differences in the strength of the associations between type of maltreatment and recidivism. In order to examine the relative impact of each predictor, hierarchical logistic regression analyses were carried out separately for sexual offending and violent offending as dependent variables. In the first step, ethnicity, gender and age were included, and in the second step abuse history was included.

## Results

In order to answer the first research question, whether there are gender differences in type of abuse experienced, the percentages of sexual abuse, physical abuse and neglect were compared between girls and boys. Percentages, *χ* ^2^ statistics indicating the differences between the groups are presented in Table 1. In order to facilitate the interpretation of the magnitude of the differences between the groups, the effect sizes in terms of Cohen’s d’s are also presented. Table 1 shows that all forms of abuse (sexual, physical abuse, neglect, and any form of maltreatment) were more often present in female than in male juvenile offenders. Additionally, the mean number of each different form of maltreatment was compared by means of a t- test, with the mean number of maltreatment incidents as dependent variable, and gender as a grouping variable. The mean number of maltreatment incidents was significantly higher in female than in male juvenile offenders (*t*(1, 13.612) = 28.60, *p* < .001.)

**Table 1:** Prevalence of sexual and physical abuse and neglect separately for female and male juvenile offenders

	Girls (*n* = 3,502)	Boys (*n* = 10,111)	*χ* ^2^/*t*	*d*
History of victimization of sexual abuse (total)	33 %	8 %	1336.76***	.66
- By a family member (SAF)	14 %	4 %	459.51^***^	.33
- Outside the family (SAOF)	24 %	5 %	1021.95^***^	.57
History of victimization of physical abuse (total)	36 %	24 %	203.16^***^	.25
- By a family member (PAF)	31 %	21 %	137.89^***^	.20
- Outside the family (PAOF)	10 %	5 %	111.90^***^	.18
History of victimization of neglect	29 %	21 %	108.77^***^	.18
Any form of maltreatment	61 %	37 %	631.16^***^	.44
Average number of forms of maltreatment	1.08 (SD = 1.12)	.55 (SD = .85)	28.60^***^	.57

^***^ *p* < .001

In order to answer the second research question, whether there are gender differences in the type of offenses committed, the percentages of sexual and violent behavior were compared for female and male juvenile offenders. The percentages and *χ* ^2^ scores are presented in Table 2. Table 2 shows that male juvenile offenders more often committed sexual aggressive behavior. The number of felony sexual offenses, misdemeanor sexual offenses and reports of sexual aggression not included in criminal history were higher in the group of male juvenile offenders than in female juvenile offenders. There were no differences between male and female juvenile offenders in the total number of violent behaviors. However, male juvenile offenders more often committed felony against person offenses than female juvenile offenders, but female juvenile offenders more often committed misdemeanor against person offenses than male juvenile offenders and more often had no reports of violence included in criminal history. The d’s indicate generally large differences between males and females in sexually aggressive behavior with Cohen’s d ranging between .76 and 1.08, and generally small differences between males and females in violent behavior, ranging between .02 (non-significant) and .35 (significant).

**Table 2:** Prevalence of sexual and violent offending behavior separately for female and male juvenile offenders

	Female (*n* = 3,502)	Male (*n* = 10,111)	*χ* ^2^	*d*
Sexual aggressive behavior (total)	3 % (*n* = 119)	10 % (*n* = 1,016)	150.53^***^	.78
- Felony sexual offense (s)	1 % (*n* = 31)	5 % (*n* = 548)	131.34^***^	1.08
- Misdemeanor sexual offense (s)	1 % (*n* = 20)	3 % (*n* = 308)	67.77^***^	1.02
- Report (s) of sexual aggression not included in criminal history	2 % (*n* = 76)	6 % (*n* = 637)	89.38^***^	.76
Violent behavior (total)	72 % (*n* = 2,522)	73 % (*n* = 7,384)	1.35	.02
- Felony against-person offense (s)	12 % (*n* = 430)	18 % (*n* = 1,840)	65.59^***^	.35
- Misdemeanor against-person offense (s)	44 % (*n* = 1,522)	37 % (*n* = 3,776)	39.34^***^	.17
- Report (s) of violence not included in criminal history	58 % (*n* = 2,030)	54 % (*n* = 5,442)	18.05^***^	.10

^***^ *p* < .001

Finally, in order to examine the associations between maltreatment and sexual and violent offending, correlations between abuse history and sexual and violent offending were compared for male and female juvenile offenders. To examine gender differences in these associations, the correlation analyses were also conducted for males and females separately. The correlations and Fischer Z scores are presented in Table 3.

**Table 3:** Correlation between different forms of maltreatment and offending behavior separately for female and male juvenile offenders

	Sexual offending			Violent offending		
	Female (*n* = 3,502)	Male (*n* = 10,111)	*Z*	Female (*n* = 3,502)	Male (*n* = 10,111)	Z
Sexual abuse (total)	.10^***^	.25^***^	7.91^***^	.08^***^	.10^***^	1.03
SAF	.07^***^	.18^***^	5.70^***^	.07^***^	.09^***^	1.03
SAOF	.10^***^	.20^***^	5.22^***^	.06^***^	.07^***^	.51
Physical abuse (total)	.03	.07^***^	2.04^*^	.15^***^	.17^***^	1.05
PAF	.02	.07^***^	2.55^**^	.15^***^	.17^***^	1.05
PAOF	.03	.04^***^	.51	.08^***^	.08^***^	0
Neglect	.03	.04^***^	.51	.03	.10^***^	3.59^***^
AM	.06^***^	.10^***^	2.05^*^	.10^***^	.16^***^	3.11^***^
Number of different forms of maltreatment	.09^***^	.15^***^	3.10^**^	.14^***^	.19^***^	2.62^**^

^*^ *p* < .05, ^**^ *p* < .01, ^***^ *p* < .001; *SAF* = Sexual abuse by a family member; *SAOF* = Sexual abuse outside family; *PAF* = Physical abuse by a family member; *PAOF* = Physical abuse outside the family; *AM* = Any form of maltreatment

Table 3 shows that a history of sexual abuse was associated with sexual offending, and that a history of physical abuse was related with violent offending. Moreover, the relation between abuse history and sexual offending was stronger in male than in female offenders, with the exception of abuse outside the family. There were no differences between males and females in abuse outside the family and neglect. For the relation between history of maltreatment and violent offending, there were less differences between male and female juvenile offenders. Only the relations between violent offending and history of neglect, any form of maltreatment and number of different forms of maltreatment were stronger in male juvenile offenders than in female juvenile offenders. In order to examine the relative impact of the abuse history, logistic regression analyses predicting sexual and violent offending were conducted, controlled for ethnicity, gender and age. Results are presented in Table 4, which shows that for predicting sexual offending, the interactions between gender and sexual abuse by a family member (SAF) and outside the family (SAOF) were significant. We conducted logistic regression analyses separately for males and females in order to interpret this interaction. These results showed that for both males and females sexual offending was predicted by history of sexual abuse by a family member and outside the family, but that the odds ratios were larger in males (*OR(SAF)* = 5.27; *OR(SAOF)* = 5.50), than in females *(OR(SAF)* = 2.66; *OR(SAOF)* = 2.283).

**Table 4:** Logistic regression analyses predicting sexual and violent offending, including gender*abuse history interactions

	Sexual offending			Violent offending		
	B	SE (B)	OR	B	SE (B)	OR
Ethnicity (white = 1)	.23**	.07	.795	-.18***	.04	1.202
Age	-.10***	.02	.903	-.08***	.01	.926
Gender (male = 1)	2.59***	.57	.075	.88*	.05	.415
History of victimization of sexual abuse (total)						
SAF	1.66***	.13	.190	.56**	.17	.569
SAOF	.171***	.12	.182	.54***	.14	.583
History of victimization of physical abuse (total)						
PAF	.14	.11	.874	.64***	.09	.529
PAOF	.15	.16	1.160	.34*	.14	.712
History of victimization of neglect	.03	.10	1.031	.02	.10	1.024
Any form of maltreatment	-.10	.12	.909	.17	.14	1.185
Gender*SAF	.69**	.26	1.995	.46*	.21	1.579
Gender*SAOF	.88**	.26	2.410	.34	.18	1.405
Gender*PAF	.13	.25	1.134	.02	.14	1.017
Gender*PAOF	-.52	.31	.592	-.02	.20	.984
Gender*Neglect	.08	.25	1.079	-.12	.14	.887
Gender*AM	.03	.33	1.026	-.14	.17	.874

^**^ *p* < .01, ^***^ *p* < .001; *SAF* = Sexual abuse by a family member; *SAOF* = Sexual abuse outside family; *PAF* = Physical abuse by a family member; *PAOF* = Physical abuse outside the family; *AM* = Any form of maltreatment. Variable number of different types of maltreatment appeared redundant and has therefore been excluded from these analyses

For the prediction of violent offending, only one gender*abuse history interaction was significant, namely for history of sexual abuse by a family member (SAF). Separate logistic regression analyses for males and females indicated that in males violent offending was predicted by histories of physical abuse by a family member *(OR(PAF)* = 1.89), physical abuse outside the family *(OR (PAOF)* = 1.41), sexual abuse by a family member *(OR(SAF)* = 1.76) and sexual abuse history outside the family *(OR(SOAF)* = 1.72), whereas in females violent offending was predicted by history of physical abuse by a family member *(OR(PAF) =* 1.87) and physical abuse outside the family *(OR(PAOF)* = 1.43).

## Discussion

The present study aimed to examine gender differences in the associations between abuse and neglect and sexual and violent offending in juvenile delinquents. Despite the numerous studies examining the relation between maltreatment and delinquency, few studies examined this relation between the different maltreatment forms (sexual, physical abuse, neglect, general maltreatment) and delinquency separately for female and male juvenile offenders, while this is important to be able to provide treatment that is responsive to the criminogenic needs of the juvenile offenders.

This study revealed that female juvenile delinquents were more often victim of sexual and physical abuse and had more often a history of neglect than male juvenile offenders. Additionally, male juvenile offenders more often committed sexual offenses and felony offenses against persons, whereas female juvenile offenders more often committed misdemeanor offenses against persons and more often had reports of violence that were not included in criminal history. Finally, a history of sexual abuse was mainly related to sexual offending, whereas a history of physical abuse was mainly related to violent offending. Inspection of gender differences indicated that for sexual offenses, the association between abuse history and sexual offenses was stronger in males than in females. For violent offenses it turned out that violence was predicted by any kind of abuse history in males, whereas only physical abuse histories significantly predicted violent behavior in females.

The finding that female juvenile delinquents were more often victim of sexual and physical abuse and more often had a history of neglect and maltreatment than male juvenile offenders has been explained by Wellman 1993. Wellman argued that females have been socialized to be more compliant and responsive to the needs of others, which may result in higher levels of abuse victimization. Alternatively, Sundaram et al. (2008) explain the different prevalence rates by possible underreporting of sexual abuse victimization by males, which may not be the case for neglect and physical abuse. This might explain why previous research found that boys, rather than girls, were more often targets of neglect and physical abuse (Sickmund et al. 1997). One should bear in mind, however, that the present study examined a sample of juvenile delinquents. The comparison with a community sample may, therefore, not be useful.

Additionally, male juvenile offenders more often committed sexual offenses and felony offenses against persons. This is in line with previous research showing higher numbers of sexual offenses by male juveniles (Finkelhor et al. 2009) and the generally lower rate of delinquency in females (e.g., Fitzgerald et al. 2012). This may mean that females express themselves differently; they may have a different coping strategy than males. For example, Adams et al. (2013) compared psychiatric problems and trauma exposure in delinquent and non-delinquent adolescents. Apart from the higher levels of trauma exposure in the delinquent sample, he reported an increased likelihood of a major depressive episode in girls in the delinquent group, suggesting females may deal with the consequences of abuse and neglect by internalizing, whereas boys are more likely to externalize.

The finding that female juvenile offenders more often committed misdemeanor offenses against persons and more often had reports of violence that were not included in criminal history can be explained by the fact that female perpetrators are more often referred to treatment than to correctional facilities (Lodewijks et al. 2008; Pajer 1998). These findings may indicate that females do, in fact, commit violence, but are less often sentenced in penal law. A finding that may confirm this hypothesis is the finding that abused and neglected female juvenile offenders more often committed violence (Gammelgård et al. 2012; Widom and White 1997).

Another possible explanation for gender differences in the association between abuse victimization and delinquent behavior is that the long-term consequences may depend on the type of abuse or neglect experienced. As females are more likely to be sexual abuse victims than males (Zahn-Waxler 1993), this may explain gender differences in associations between abuse type and delinquent behavior. Victims of neglect and physical abuse are believed to have the largest risk to show delinquent behavior, whereas this risk is not present in sexual abuse victims (Stewart et al. 2002). Additionally, in line with, this Trickett and McBride-Chang (1995) suggested victims of physical abuse were more likely to show externalizing problems. Victims of sexual abuse were more likely to show internalizing behavior problems. Another explanation for (gender) differences in the association between abuse victimization and delinquent behavior can be found in the individual differences in coping ability. Individual differences do affect the way a person copes with maltreatment. Females are, for example, twice as likely to develop post-traumatic stress disorder (PTSD) (Koenen and Widom 2009). Among detained juveniles substantial percentages of boys and girls experience posttraumatic symptoms, with a higher percentage of girls suffering from post-traumatic stress symptoms (Kerig and Becker 2011). Becker and Kerig 2011 further showed that the severity of post-traumatic stress symptoms, in turn, predicts the degree of delinquency. So, the differences in trauma may also explain (gender) differences in the strength of the association between maltreatment victimization and delinquent behavior.

Finally, a history of sexual abuse was mainly related to sexual offending, whereas a history of physical abuse was mainly related to violent offending. These findings suggest that in a group of juvenile offenders, there are indications for a cycle of violence. This further supports the assumption that children who experienced physical abuse are at increased risk for developing violent behavior (Widom 1989b). It should be noted, however, that the cycle of violence may oversimplify what actually happens with abuse victims. The cycle of violence states that maltreated children have an increased likelihood to exert violence themselves. However, only being an abuse victim will never be the only explanation of the development of delinquent behavior. The majority of survivors do not go on to be violent, especially not sexual abuse victims (Collishaw et al. 2007). Previous research has demonstrated that delinquent behavior is multi-determined, indicating that dysfunctional development is never caused by just one adverse situation or risk factor, but that it is always an interaction of various risk factors (in the child itself, and in the broader social context of the child) which causes the development of violent delinquent behavior (see for example Loeber et al. 2009). In fact, the finding that male and female abuse victims react differently to abuse victimization indicates that one can never conclude that all maltreatment will result in violence. However, the present study did show that both in males and females, history of abuse victimization increases the likelihood to commit violent offenses. Potential gender differences need further study.

The present study further specifies the association between maltreatment and violence by indicating that sexual offenders more often have a history of sexual abuse, whereas violent offenders have a history of abuse in general. These findings confirm social learning theories, assuming that victims copy behavior they have learned (Bandura 1977). Other studies also reported that experiencing a particular type of maltreatment is most likely to result in the same type of offending behavior (Hamilton et al. 2001). Dutton and Hart 1992 also found an association between physical abuse and violent offending and an association between sexual abuse and sexual violence. However, remarkably, Zingeraff et al. 1993 found that maltreatment was mainly related to status offenses, such as runaway, and not with violent behavior.

It seems that, in a group of juvenile offenders, the cycle of violence is more present in males than in females. In other words, and in line with the work of Fagan 2005, the association between childhood abuse and offending behavior appears to be stronger in males than in females. When examining the association between abuse history and violence, in males, sexual abuse history was predictive, which was not the case in females showing violence. If committing crimes is considered to be a coping mechanism, this suggests a different coping mechanism in males and females. In this context, Ford et al. (2013) recently showed that there is a small subgroup of what they call “poly-victims” among the juvenile detention population, mainly girls, who have experienced several types of traumatic victimization, and who needed attention because of their severe behavioral and mental risks. Females who have been abuse victims thus need attention in preventive and curative interventions. Ford et al. (2011) showed that abuse victims generally deserve attention because of their increased risk for exerting violence, and because their abuse history is an indication of problem severity. For abused males, the link between abuse victimization and delinquent behavior should further be examined in order to develop adequate preventive and curative interventions, likely incorporating treatment of the traumas present (Kerig and Becker 2010).

The present study is, to our knowledge, the first examining gender differences in the association between different kinds of offending behavior and histories of maltreatment. Generally, few gender differences in the association between abuse victimization and offending behavior have been found. The present study thus indicates that in both male and female juvenile offenders maltreatment history is associated with offending behavior, suggesting that in facilities for juvenile delinquents, attention should be given to possible abuse histories of offenders. Various theories explain the link between maltreatment victimization and offending (e.g., theories based on disturbed attachment in juvenile delinquents or theories on coping with trauma, explaining delinquent behavior from emotion dysregulation; Kerig and Becker 2010). These theories may be the starting point for preventive or curative interventions, especially for males who have been sexually abused and deserve attention because they are likely to pose the greatest risk, as they tend to commit both sexual and violent offenses.

### Limitations

First, it is important to be aware that the information on maltreatment used in the present study is retrospective in nature; specifically, when juveniles were accused of delinquent behavior, their maltreatment history was identified. This may have affected the results, because their recent justice contact may have affected their perception of their childhood experiences. Additionally, it should be noted that maltreatment scores were based on suspected maltreatment, whether or not substantiated. This may have resulted in higher rates of abuse than the actual numbers.

Second, it is important to realize that maltreatment in the present study was examined in a group that was referred to court for offenses. This may have resulted in an increase in the strength of the relation between maltreatment and delinquent behavior (Zingeraff et al. 1993). Findings cannot, therefore, be generalized to community samples. The association between maltreatment and offending may not be present or may be less present in a community sample, because it is likely that only a (very) small part of all abuse victims become violent (e.g., Stith et al. 2000). Future research may replicate this study in a community sample, to find out whether there are differences between a community and juvenile justice sample in the strength of the association between maltreatment history and delinquent behavior. Ford et al. (2013) compared a ‘vulnerable’ and a ‘non vulnerable’ population and concluded that youth in the juvenile justice setting all experienced substantial victimization comparatively.

Additionally, other important factors that may have affected abuse resiliency, such as developmental trauma (Leibowitz et al. 2012), psychiatric diagnoses (e.g., PTSD) academic performance, or IQ, and offending behavior have not been included in this study. Kerig and Becker 2010 suggested various theoretical models explaining the association between abuse history and the development of delinquent behavior. More comprehensive models may be needed to further explain the findings of the present study. Future research, including these constructs, will be needed to obtain more complete knowledge on gender differences in the association between abuse victimization and offending.

### Conclusions and practical implications

Notwithstanding these limitations, there are some important conclusions that can be drawn from this study. First, the present study supported previous findings that female juvenile delinquents are victim of sexual and physical abuse more often than male juvenile offenders. Society should take action to identify these victims earlier and to protect them against maltreatment. Given the higher levels of maltreatment experienced by females, it is important to give attention to Post Traumatic Stress Disorder (PTSD) in interventions for female juvenile delinquent females (Gammelgård et al. 2012), as the delinquent behavior is possibly related to consequences of the experienced abuse, such as PTSD (Kerig and Becker 2010).

In terms of prevention of delinquent behavior, it is important to be aware that male juvenile offenders commit more often sexual offenses and felony offenses against persons, whereas female juvenile offenders more often commit misdemeanor offenses against persons and more often have reports of violence that are not included in criminal history. Youth care providers should be aware that females may be less likely to have a formal criminal record, but may still have committed violence. Given the association between maltreatment and delinquent behavior for both boys and girls, interventions could pay attention to potential effects of maltreatment and to the assessment of abuse and neglect histories in juvenile delinquents. The finding that sexual abuse is mainly related to sexual offending and history of physical abuse is mainly related to violent offending provides targets for prevention initiatives.

Future research should focus on dynamic risk factors that are related to sexual and violent offending. Moreover, future research should examine the consequences of abuse victimization also in non-offender groups, in order to gain a more complete picture of the consequences of maltreatment. Generally, the relations between victimization and offending are stronger in male juvenile offenders than in female juvenile offenders, especially the relation between sexual abuse and sexual offending. The consequences of abuse victimization are different for juvenile males then females, in particular with respect to delinquent behavior. Interventions targeting the prevention of criminal offense recidivism should, therefore, pay particular attention to the negative consequences of abuse victimization in male juveniles.

## Appendix 1

Section of the manual used to assess abuse histories of juvenile offenders: (Washington State Institute for Public Policy 2004, page: 98):

**Table:**

2. History of physical abuse: Check with child protective services, community mental health, and other sources for information.	Has an adult ever physically hurt you? Tell me what happened. How often? Last time?
3. History of sexual abuse: Check with child protective services, community mental health, and other sources for information.	Has anyone ever touched you in a way that made you feel uncomfortable? Who was it? How often? Last time? Did you ever feel someone was trying to take advantage of you sexually? Insisting on sexual activity? Who was it?
4. History of being a victim of neglect: Check with child protective services, community mental health, and other sources for information.	Child Protective Services defines neglect to include negligent or maltreatment (dangerous act) or omission that constitutes a clear and present danger to the child’s health, welfare, and safety. Have you always been taken care of, given enough to eat?”
